# Supplementary material for: c-Kit modifies the inflammatory status of smooth muscle cells
Source: PeerJ. 2017 Jun 13;5:e3418. doi: 10.7717/peerj.3418 (PMC5472039; doi:10.7717/peerj.3418)
Supplement: Table S1 [file peerj-05-3418-s001.docx]

| **Supplementary Table 1. Knockdown of TAK1, NLK and GFP (negative control) in Kit^+/+^ smooth muscle cells by siRNA** | |
| --- | --- |
|  |  |
| **Target** | **siRNA Pool Sequences** |
|  | 771 5’- GCCCATTGAGAGCTTGATGACACGCTGTT -3’ |
| TAK1 | 880 5’- GCGGATGAGCCGTTACAGTATCCTTGTCA -3’ |
|  | 1073 5’- AGCAACAGAGTGAATCTGGACGCCTGAGC -3’ |
|  | 1474 5’- TCAGATAACTCCATCCCAATGGCGTATCT -3’ |
|  | 447 5’- TGGCGTTGTCTGGTCAGTAACAGATCCAA -3’ |
| NLK | 892 5’- ACTCAGGAAGTAGTTACTCAGTATTACCG -3’ |
|  | 1103 5’- CTTGTGAAGGTGCTAAGGCACACATACTC -3’ |
|  | 1477 5’- AGAGTGCCTCTCTGCATCAACCCGCAGTC -3’ |
| GFP | 5’- GGGTGAACTCACGTCAGAA -3’ |
