# Supplementary figures and images for: c-Kit modifies the inflammatory status of smooth muscle cells
Source: PeerJ. 2017 Jun 13;5:e3418. doi: 10.7717/peerj.3418 (PMC5472039; doi:10.7717/peerj.3418)

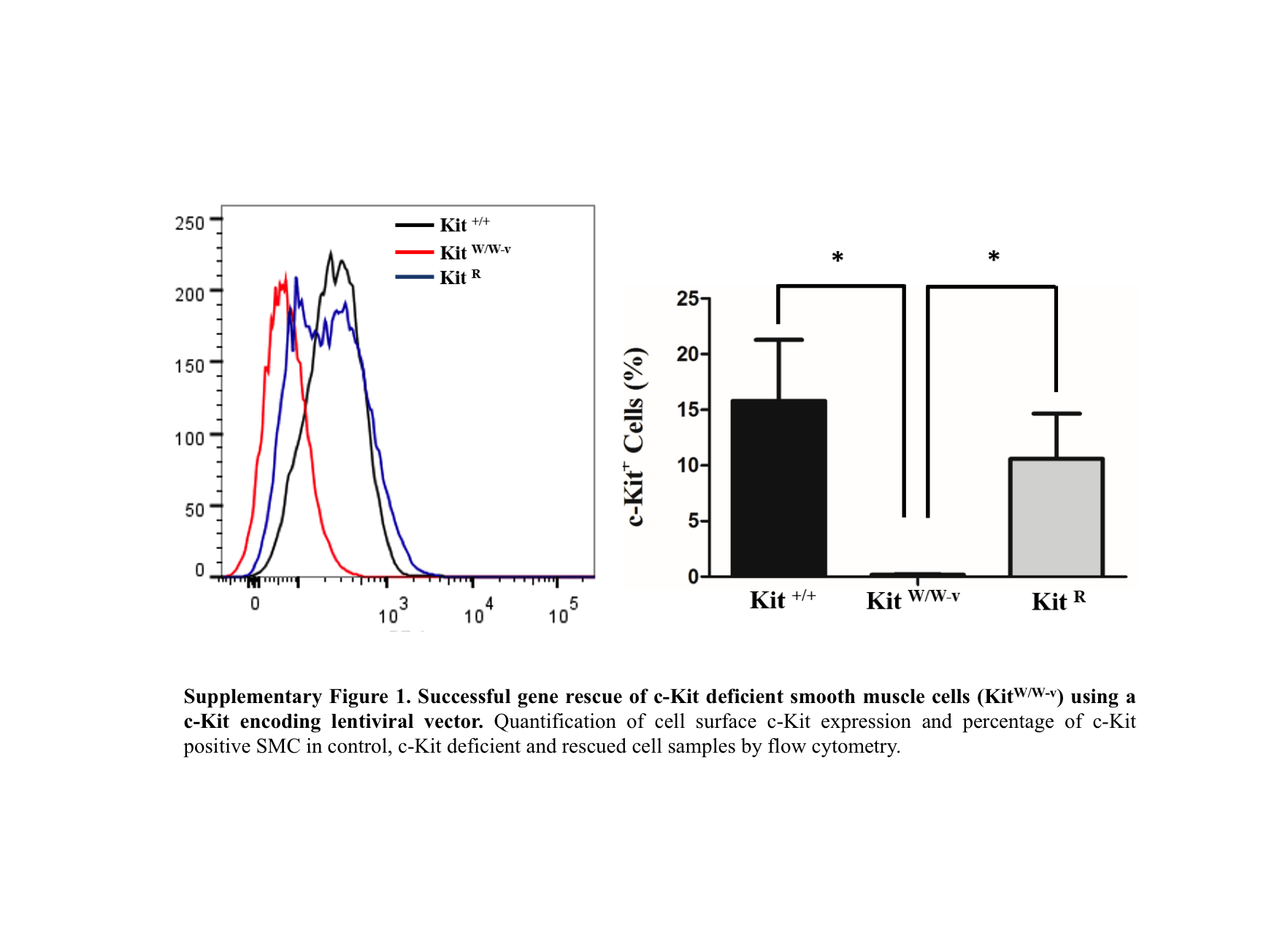

Supplement: Figure S1 — Quantification of cell surface c-Kit expression and percentage of c-Kit positive SMC in control, c-Kit deficient and rescued cell samples by flow cytometry. [file peerj-05-3418-s002.png]

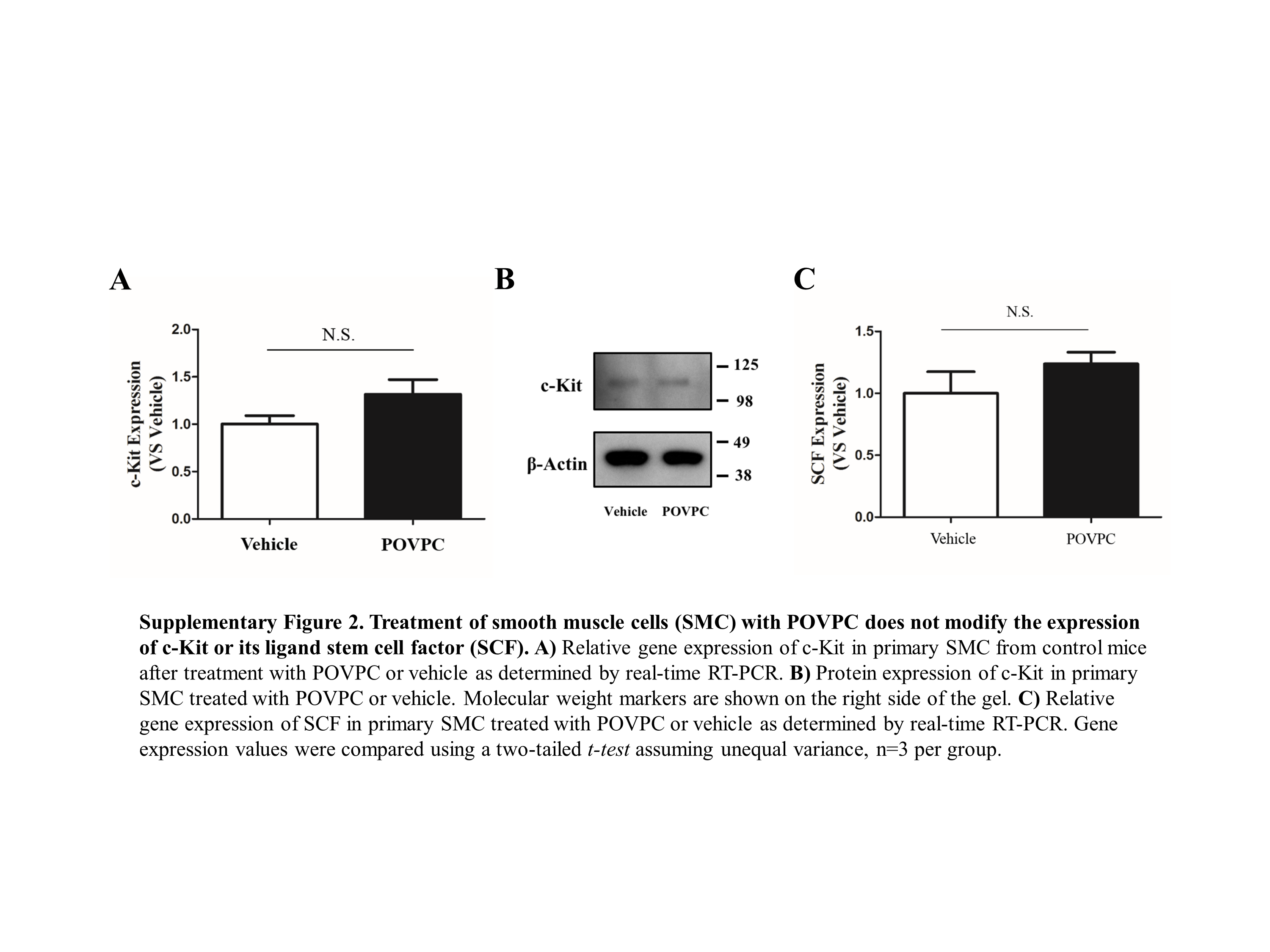

Supplement: Figure S2 — (A) Relative gene expression of c-Kit in primary SMC from control mice after treatment with POVPC or vehicle as determined by real-time RT-PCR. (B) Protein expression of c-Kit in primary SMC treated with POVPC or vehicle. Molecular weight markers are shown on the right side of the gel. (C) Relative gene expression of SCF in primary SMC treated with POVPC or vehicle as determined by real-time RT-PCR. Gene expression values were compared using a two-tailed t-test assuming unequal variance, n = 3 per group. [file peerj-05-3418-s003.png]

## Slide 1
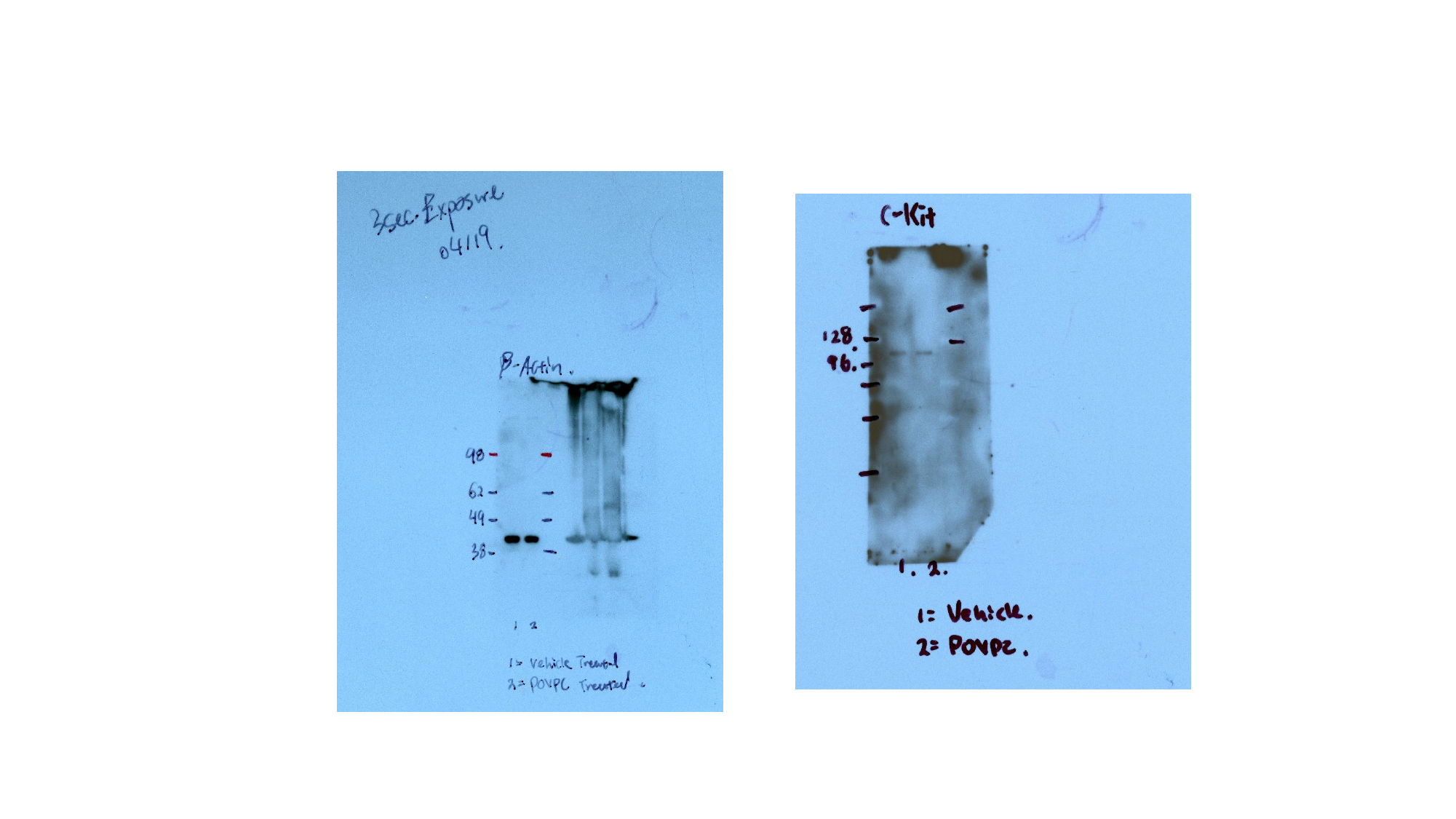

Supplement: Supplemental Information 9 [file peerj-05-3418-s012.pptx]
