# Supplementary material for: c-Kit modifies the inflammatory status of smooth muscle cells
Source: PeerJ. 2017 Jun 13;5:e3418. doi: 10.7717/peerj.3418 (PMC5472039; doi:10.7717/peerj.3418)
Supplement: Supplemental Information 8 [file peerj-05-3418-s011.pptx]

## Slide 1
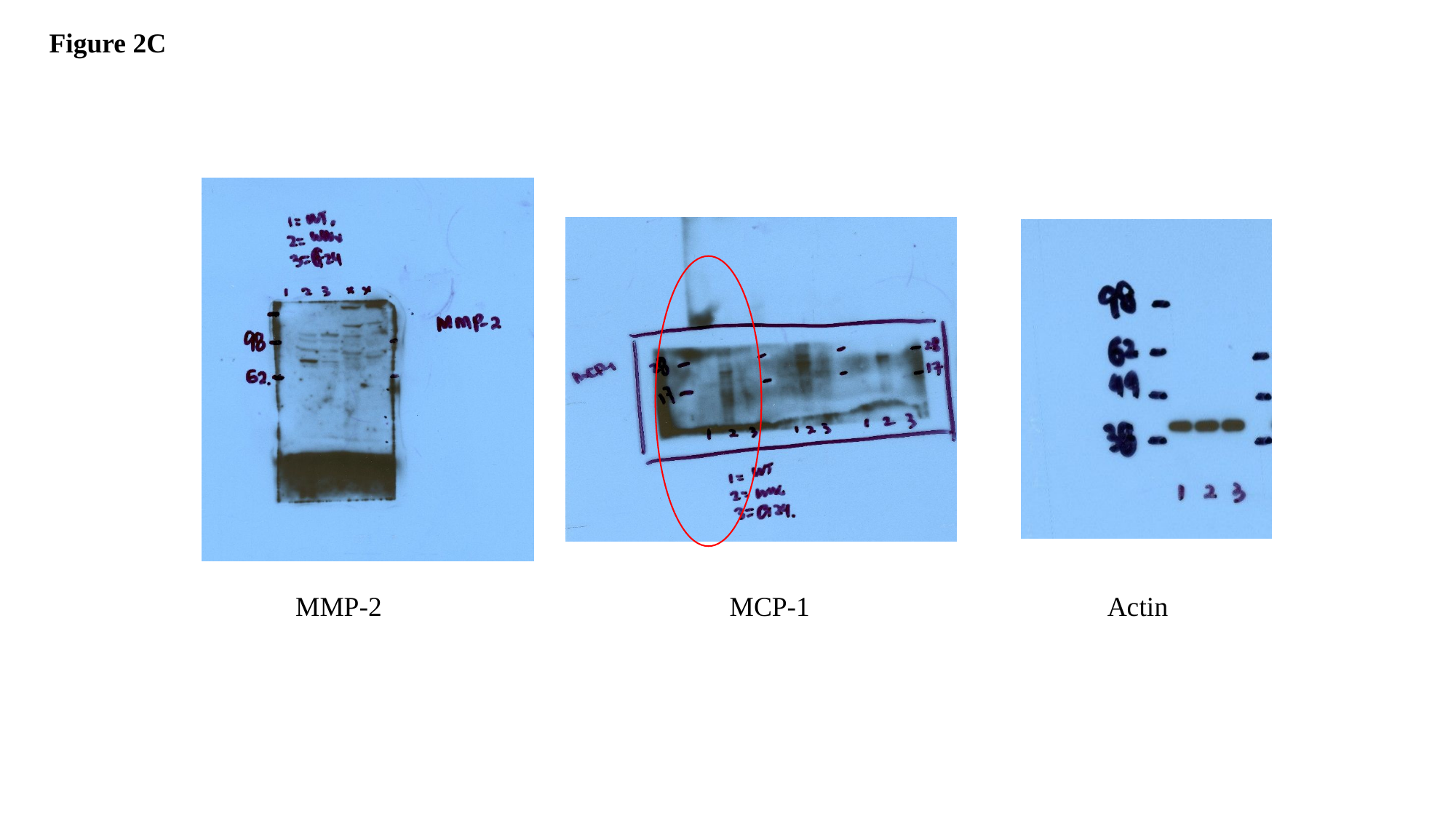

Figure 2C
MMP-2
MCP-1
Actin

## Slide 2
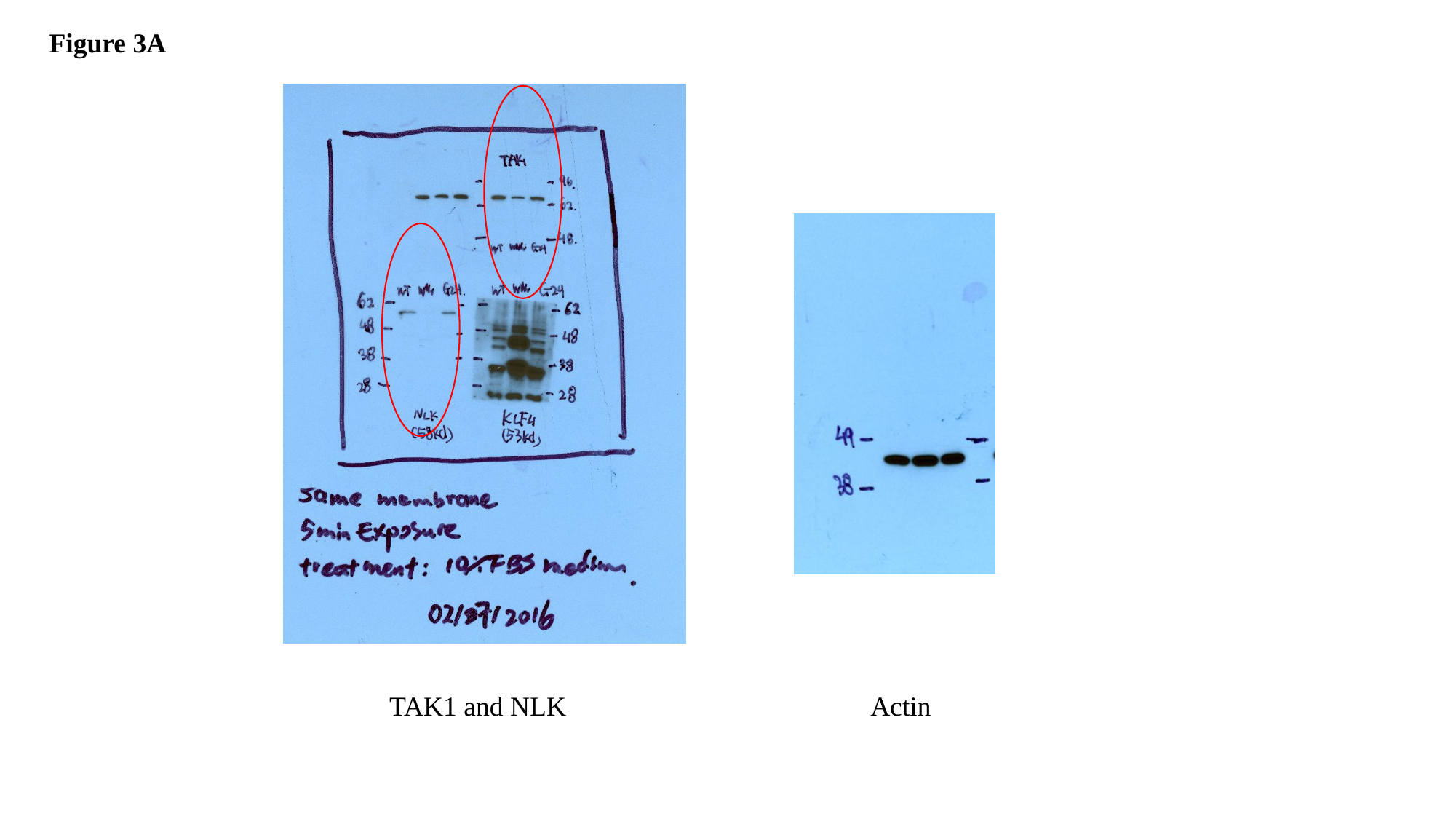

Figure 3A
TAK1 and NLK
Actin

## Slide 3
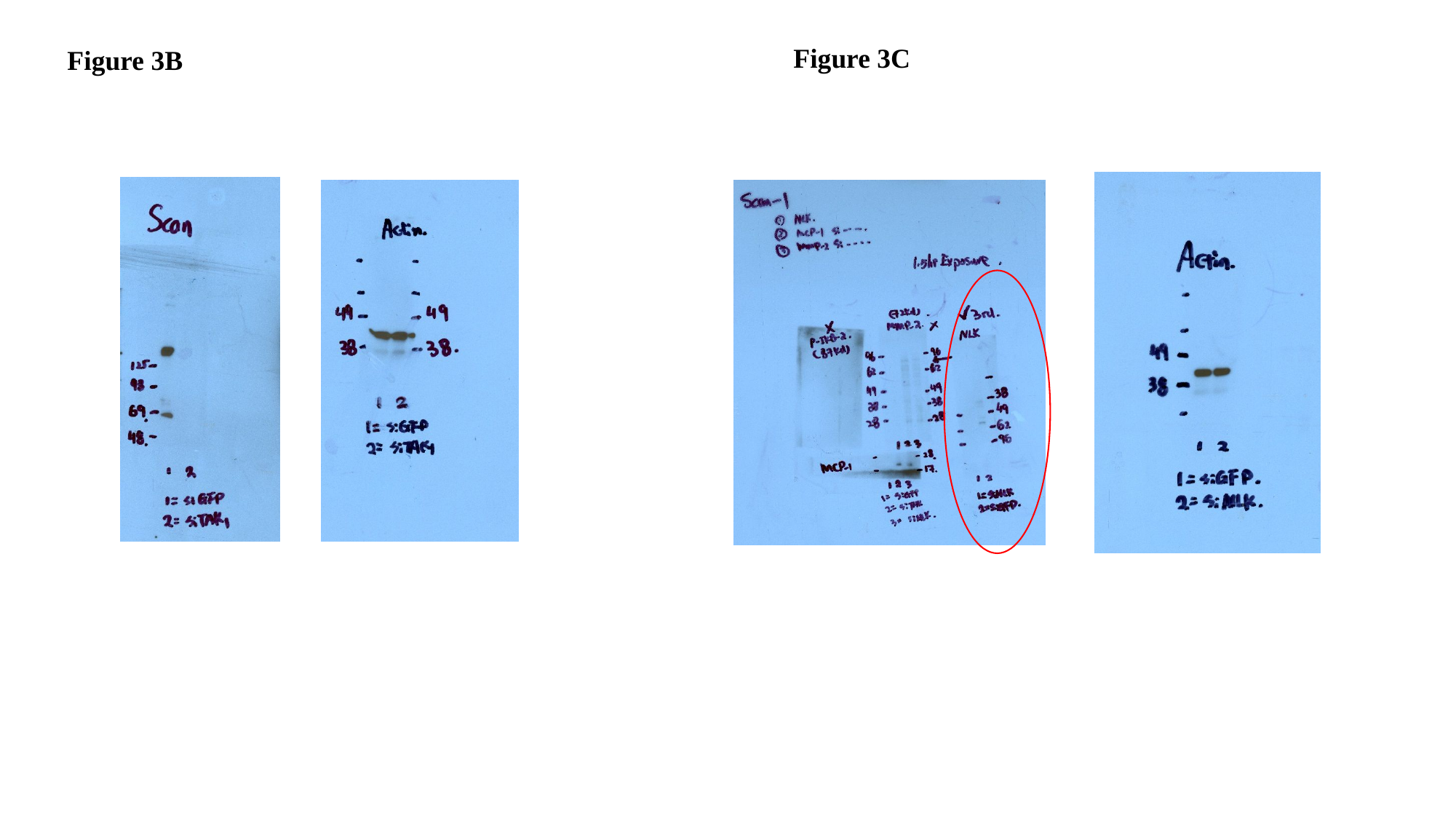

Figure 3C
Figure 3B

## Slide 4
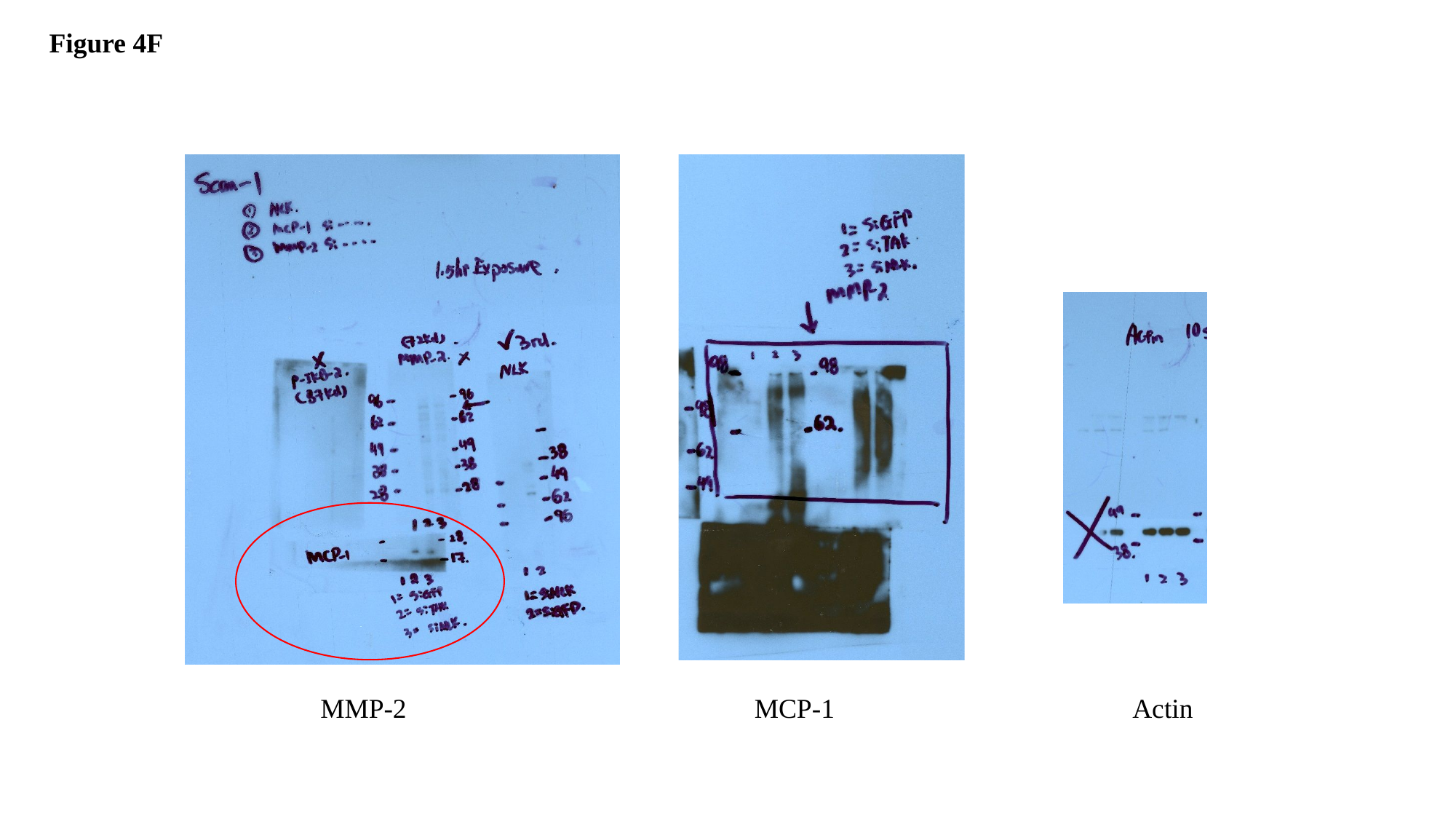

Figure 4F
MMP-2
MCP-1
Actin

## Slide 5
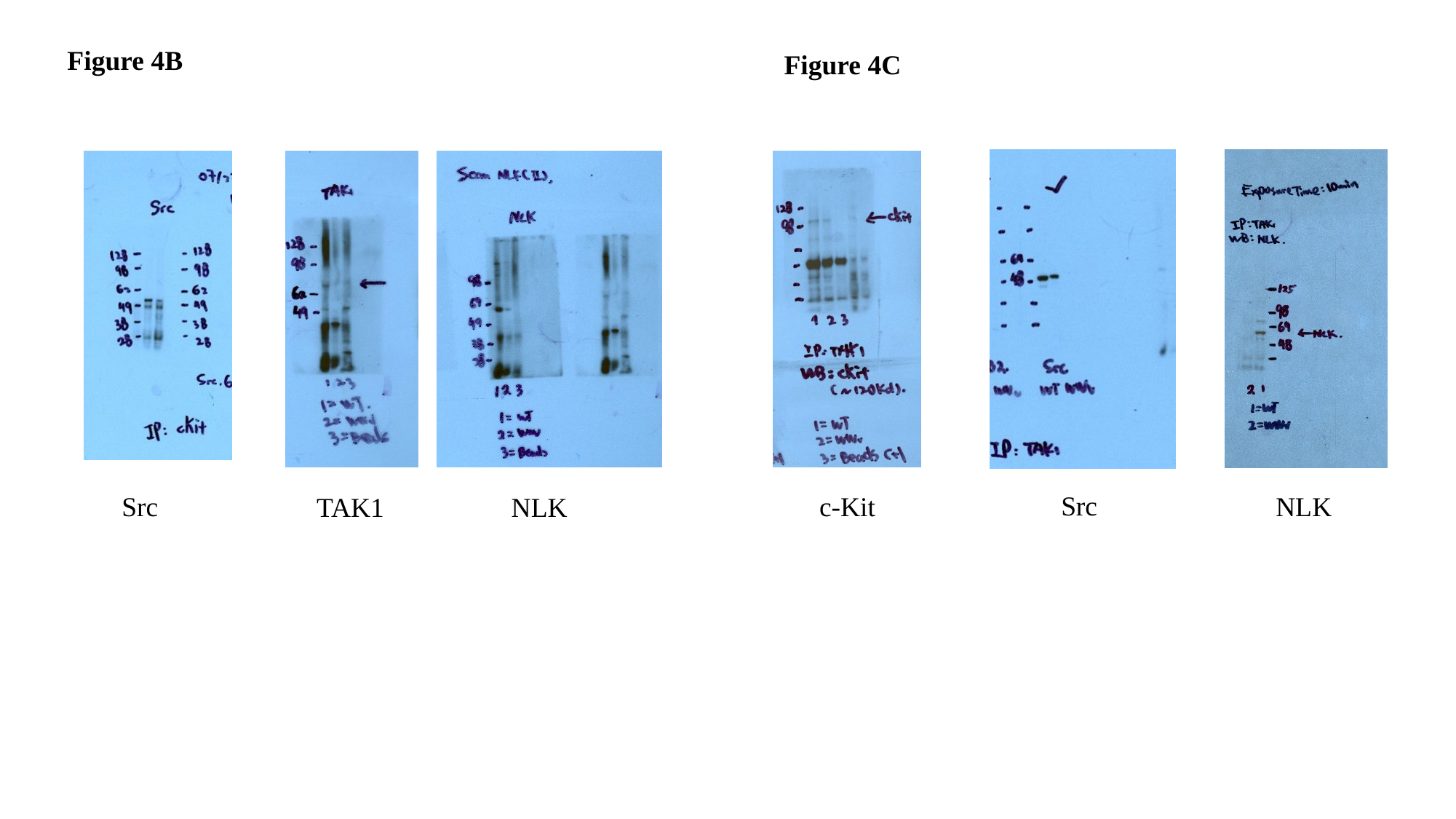

Figure 4B
Figure 4C
Src
Src
c-Kit
NLK
NLK
TAK1
